# Working with alcohol prevention in occupational health services: “knowing how” is more important than “knowing that” – the WIRUS OHS study

**DOI:** 10.1186/s13722-022-00335-0

**Published:** 2022-10-01

**Authors:** Mikkel Magnus Thørrisen, Talieh Sadeghi, Tore Bonsaksen, Ian D. Graham, Randi Wågø Aas

**Affiliations:** 1 Department of Rehabilitation Science and Health Technology, Faculty of Health Sciences, OsloMet – Oslo Metropolitan University, Oslo, Norway; 2 Department of Public Health, Faculty of Health Sciences, University of Stavanger, Stavanger, Norway; 3 Work Research Institute, Centre for Welfare and Labour Research, OsloMet – Oslo Metropolitan University, Oslo, Norway; 4 Department of Health and Nursing Sciences, Faculty of Social and Health Sciences, Inland Norway University of Applied Sciences, Elverum, Norway; 5 Department of Health, Faculty of Health Studies, VID Specialized University, Stavanger, Norway; 6 Centre for Practice-Changing Research, Ottawa Hospital Research Institute, Ottawa, Canada; 7 School of Epidemiology and Public Health, University of Ottawa, Ottawa, Canada

**Keywords:** Alcohol consumption, Knowledge translation, Occupational health services, Prevention, Workplace interventions, Workforce

## Abstract

**Background:**

Alcohol consumption is a major public health challenge; the majority of employees consume alcohol regularly and a considerable proportion of employees can be characterized as risky drinkers in need of interventions. Occupational health services (OHS) are uniquely positioned for implementing alcohol prevention interventions targeting employees, but rarely do so. Studies have shown that lack of knowledge among OHS personnel is a barrier to alcohol prevention activity. This study aimed to explore OHS personnels’ levels of theoretical and practical alcohol knowledge, and whether these two ways of knowing were differentially associated with alcohol prevention activity.

**Methods:**

In this cross-sectional study, survey data were collected from 322 OHS personnel in Norway in 2018 (response rate = 53.6%). The survey included variables of two ways of knowing (theoretical and practical) and three types of doing (intervention frequency, conducting individual interventions, and conducting group interventions). Data were analyzed with descriptive statistics, paired sample t-tests, bivariate correlations, and adjusted linear and logistic regression analyses.

**Results:**

OHS personnel rated their theoretical alcohol knowledge higher than their practical knowledge (η^2^ = 0.33, *p* < 0.001). Higher reported levels of practical knowledge were associated with higher intervention frequency (b = 0.39, β = 0.60, *p* < 0.001) and greater likelihood of conducting individual interventions (OR = 1.60, *p* < .001) as well as group interventions (OR = 1.84, *p* < 0.001). Theoretical knowledge was not associated with conducting interventions, and there was no evidence of an interaction between the two ways of knowing in their association with doing. Sensitivity analyses did not indicate clustering effects of OHS personnel being employed within different units.

**Conclusions:**

Different ways of knowing about alcohol among OHS personnel were dissimilarly associated with conducting alcohol prevention interventions in occupational health settings. For doing, knowing how seems to be more important than knowing that. Training programs for OHS personnel should emphasize knowledge about how to deal with alcohol-related issues and how to conduct prevention interventions, rather than focus on detrimental effects of alcohol.

**Supplementary Information:**

The online version contains supplementary material available at 10.1186/s13722-022-00335-0.

## Introduction

Harmful alcohol consumption represents a global public health challenge associated with disease, disability and mortality [1–7], as well as with injuries and violence [8–12]. Harmful alcohol consumption comprises different aspects of alcohol use, including high average consumption volumes (e.g., grams per day/week/month) [1–5, 7, 10, 12], heavy episodic drinking (i.e., (ir)regular binge drinking episodes) [2, 4], acute use of alcohol in high-risk situations (e.g., while driving) [8, 9, 11], and drinking patterns that have resulted in alcohol-related diagnoses (e.g., alcohol use disorders) [6]. A majority of employed adults consume alcohol regularly [13], and one to three out of ten employees have been found to consume alcohol at a risky level and may benefit from alcohol prevention interventions [14]. Risky drinking constitutes a drinking pattern that increases the risk of medical, social, legal, occupational, domestic and economic problems [15]. The majority of society’s risky drinkers are part of the active workforce [16], and alcohol consumption among employees is associated with higher levels of sickness absenteeism [17–20] and presenteeism (impaired on-the-job performance) [21–23].

Reducing harmful alcohol consumption has been emphasized as a keystone in the United Nations’ sustainable development goal of health [24, 25], and there is evidence that brief alcohol prevention interventions targeting employees have favorable effects [16, 26, 27]. The workplace constitutes, according to the World Health Organization [28], a priority setting for health promotion and illness prevention. Occupational health services (OHS) aim to protect and promote employees’ safety and health, prevent productivity loss, and improve working conditions [29]. Estimations of OHS coverage imply that such services have the potential to reach a large proportion of employees (e.g., France: 90%, Finland: 85%, Italy: 80%, Norway: 60%, USA: 35%) [29]. Aimed at early identification of illness and adverse lifestyle outcomes, OHS routinely perform health examinations of employees. Studies from Sweden [30] and the UK [31] suggest that such examinations are received positively by employees. Trials have reported that alcohol prevention activities may appropriately and feasibly be integrated in OHS’ routine health examinations [32–34]. However, a Norwegian study [35] indicated that OHS personnel engaged in alcohol prevention quite rarely (less than on a monthly basis), even though they believed employees’ alcohol consumption to constitute a public health challenge and agreed that OHS should increase its prevention activity. Several authors have advocated that the OHS should obtain a more proactive role in alcohol prevention [36–38].

Bridging the gap between what is known and what is done, referred to as the “know-do gap”, has been emphasized as one of the most important challenges for the field of public health [39]. Clinical decisions are often not supported by scientific evidence [40]. In real world clinical settings, decisions are influenced by a variety of factors beyond research evidence, for instance social interactions between patients and health care personnel, differing health values and beliefs, and variable availability of resources [41]. Patients often do not receive the best possible practices, and in some instances they even receive potentially harmful services [42, 43].

Knowing can be distinguished from doing, and knowledge translation refers to the process of putting knowledge into action, i.e., the process of synthesizing, adapting and disseminating knowledge in order to improve health services and health outcomes [44]. The path from knowing to doing is often far from straightforward, partly due to knowledge having to be subjected to translational processes, and partly as a result of knowledge itself constituting a multifaceted phenomenon. Different types of knowledge co-exist, and different types of knowledge may be dissimilarly associated with doing. A fundamental distinction has been made between theoretical (factual/propositional) knowledge (knowing that something is the case) and practical knowledge (knowing how to do something) [45]. These two ways of knowing can be distinguished by being differentially related to practical abilities [46], yet they may both be important in understanding how knowing can translate into doing. Fantl [46] emphasized that different views on the relationship between theoretical and practical knowledge have been advocated in the literature. The two ways of knowing may be conceived as fundamentally independent, insofar that the one does not require the other. In this perspective, knowing how to do something (practical knowledge) does not hinge on knowing that something is the case or why it may be important to do something (theoretical knowledge), and vice versa. On the other hand, the two ways of knowing may be perceived as interwoven. Hence, having practical knowledge about how to do something could require some degree of theoretical knowledge (e.g., by theoretical knowledge moderating the association between practical knowledge and doing), or the other way around. As postulated by expectancy-value theory [47], whether and to what extent an individual performs an activity can be explained in terms of his/her belief about how well (s)he is able to perform the activity (expectancy), and the degree to which (s)he values the activity. Hence, the action of doing may be a function of both theoretical knowledge (knowing that and thus why it is important to perform an activity) and practical knowledge (knowing how to successfully perform the activity).

Health care personnel’s level of alcohol training has been found to predict the extent to which they actually treat individuals with alcohol-related problems [48]. Although results are somewhat inconsistent, studies suggest that health care personnel have quite a low level of alcohol-related knowledge [49–54]. Similar findings have been reported for students in health professions [55, 56]. Consequently, the importance of developing training strategies aimed at improving health care personnel’s alcohol-related knowledge has been emphasized [49, 50].

Research on factors affecting alcohol prevention activity in the OHS is sparse, although some studies point to knowing as important for doing. In a Swedish study [57], OHS personnel reported that knowledge about counseling techniques was the most important facilitator for increased alcohol prevention activity. Similarly, a study of Norwegian OHS personnel [35] identified lack of alcohol-related knowledge as a significant barrier (alongside lack of time and resources). The study did not, however, differentiate between theoretical and practical alcohol-related knowledge. Knowing that alcohol consumption constitutes a public health challenge by being associated with detrimental health and occupational outcomes, and knowing how to conduct alcohol prevention interventions in a practical setting may be differentially associated with alcohol prevention activity. Stated differently: Theoretical and practical knowledge may be dissimilarly related to doing. One may hypothesize that conducting alcohol prevention interventions can be understood, at least partly, based on how OHS personnel appraise their ability to conduct such interventions (i.e., based on their expectancies, which may be related to their level of practical knowledge), and based on the extent to which they value alcohol prevention (i.e., based on their values, which may be related to their level of theoretical knowledge regarding detrimental effects of alcohol consumption). The provision of better services hinges on generating a better understanding of the know-do gap among health care personnel. In order to determine what type of knowing should be included and emphasized in training programs for health care personnel to promote conducting alcohol prevention interventions, it is important to explore whether and how the two ways of knowing are associated with doing.

### Study aims

This study aimed to explore (i) how OHS personnel self-assessed their theoretical and practical alcohol knowledge, (ii) whether and how theoretical and practical knowledge were associated with alcohol intervention frequency, and whether the two ways of knowing interacted in their relationship with intervention frequency, and (iii) whether the two ways of knowing were differentially associated with conducting alcohol prevention interventions targeting individual employees and groups.

## Methods

### Design and setting

The study was based on a cross-sectional survey among personnel in 69 OHS units in Norway. Data were collected in 2018 as part of the Norwegian national WIRUS project (Workplace Interventions preventing Risky alcohol Use and Sick leave).

### Data collection and sample

All nationally approved OHS units in Norway in 2018 (n = 206) were contacted and invited to participate in the study, based on contact information collected from the Norwegian Labor Inspection Authority. Sixty-nine units (74.2% of the responding units; 33.5% of all nationally approved units) agreed to participate and provided the researchers with e-mail addresses for all personnel in their unit. Units from all geographical counties in Norway and units providing services for companies in all work divisions (based on Eurostat’s classification of economic activities [58]) were represented among the 69 OHS units. One-hundred-and-thirteen units did not respond. Ninety-three declined to participate. Only 12 of these provided a reason for not participating (unable due to high workload: n = 9; unable due to ongoing reorganization: n = 2; perceived the study as irrelevant: n = 1).

Digital questionnaires were distributed to 601 OHS personnel in the 69 units. A total of 357 (59.4%) responded. Thirty-three were excluded due to not responding on all relevant study items, leaving a final study sample of 322 OHS personnel (response rate = 53.6%). The median number of eligible OHS personnel in each OHS unit was seven, while the median number of respondents in each unit was four. Females constituted the majority of the sample (79.2%), participants’ mean age was 48.9 years (*SD* = 10.1 years), and they had substantial experience with occupational health care (mean OHS experience = 12.0 years, *SD* = 9.1 years). Nurses (n = 122, 37.9%), physiotherapists (n = 57, 17.7%) and physicians (n = 42, 13.0%) were the most frequent professions in the sample. Study sample characteristics are presented in Table 1.

**Table 1:** Characteristics of the study sample (N = 322)

Variable		
Age (years), *M*, *SD*	48.9	10.1
OHS experience (years), *M*, *SD*	12.0	9.1
Education length, *M*, *SD*	5.2	2.0
Sex		
Male, *n*, %	67	20.8
Female, *n*, %	255	79.2
Educational background		
Nurse, *n*, %	122	37.9
Physician, *n*, %	42	13.0
Physiotherapist, *n*, %	57	17.7
Other health profession^A^, *n*, %	57	17.7
Other non-health profession^B^, *n*, %	44	13.7

^A^ E.g., Psychologist, occupational therapist, occupational hygienist, medical secretary, assistant nurse, midwife

^B^ E.g., Business/administration/management, engineer, educationalist/teacher, social worker, social scientist

It was a priori defined that a satisfactory sample size had to exceed a recommended ratio of 15 participants per predictor variable [59], as well as exceeding a minimum required sample size in accordance with the formula N > 50 + (8 × number of predictors) [60].

### Measures

#### Knowing

OHS personnel’s estimated degree of *theoretical alcohol knowledge* was based on how they rated their knowledge about the following issues: (i) knowledge about the association between alcohol and health consequences, (ii) knowledge about the association between alcohol and sickness absenteeism, and (iii) knowledge about the association between alcohol and presenteeism (impaired on-the-job performance). Each issue was rated on a discrete numerical scale ranging from 1 (no knowledge) to 11 (very much knowledge). A composite measure was constructed by calculating a mean score for the three issues combined, resulting in a theoretical alcohol knowledge scale ranging from 1 to 11 (higher score indicated higher level of knowledge).

OHS personnel’s degree of practical alcohol knowledge was measured with one item, asking respondents to rate, on a discrete numerical scale ranging from 1 (no knowledge) to 11 (very much knowledge), their knowledge about how to conduct alcohol prevention interventions.

The a priori distinction between the three theoretical issues and the practical issue was supported by a confirmatory principal component analysis. The three theoretical issues loaded on the first factor, while the practical issue loaded on the second factor [see Additional file 1].

#### Doing

*Alcohol intervention frequency*, i.e., how often OHS personnel typically conducted alcohol interventions targeting employees, was measured on a seven-point Likert scale (1 = never; 2 = less than yearly; 3 = yearly; 4 = less than monthly; 5 = monthly; 6 = weekly; 7 = daily).

Moreover, in order to differentiate between different levels of doing, OHS personnel were asked to indicate whether they regularly conducted alcohol prevention interventions targeting groups and individuals, respectively. *Group-level interventions* were coded as a dichotomous variable (0 = no; 1 = yes). A score of 1 indicated that OHS personnel regularly engaged in one or more of the following interventions: group education, general advice to managers, and general collaboration with Akan (an organization that plays an important role in handling alcohol, drug, gambling and gaming issues among employees in Norway [61]). Similarly, *individual-level interventions* were coded as a dichotomous variable (0 = no; 1 = yes), with a score of 1 indicating that OHS personnel regularly engaged in individual counseling and/or alcohol screening of employees.

**Fig. 1:**
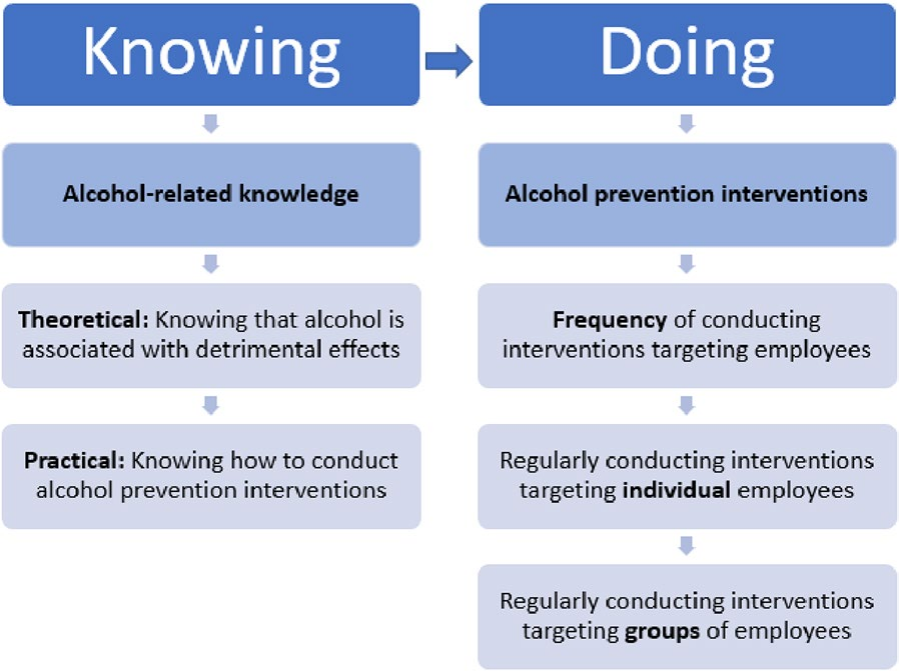
Conceptual model in this study of two ways of knowing and three ways of doing in the case of alcohol prevention in occupational health settings

A conceptual model of the two ways of knowing and three ways of doing is presented in Fig. 1.

#### Covariates

The following covariates, chosen a priori based on previous research on OHS in Norway [35, 62], were included in adjusted analyses: *sex* (male; female), *age* (years), *education length* (years of university/college education), *OHS experience* (years), OHS personnel’s *drinking attitudes*, which were measured with the Drinking Norms Scale [63] (mean score of seven items; low score = restrictive attitudes, high score = liberal attitudes), and *educational background* (nurse; physician; physiotherapist; other health profession; other non-health profession). Educational background (categorical nominal variable) was dummy coded for inclusion in adjusted analyses.

### Analysis

All analyses were performed with IBM SPSS version 27. Significant results were defined as *p* < 0.05. Selections of statistical procedures were based on variables’ level of measurement, sample size and whether tests’ assumptions were appropriately met.

#### Main analyses

The study variables were analyzed descriptively using means (*M*), standard deviations (*SD*), frequencies and percentages, as appropriate. A paired sample t-test was applied to explore the difference between OHS personnels’ levels of theoretical and practical alcohol knowledge. Eta squared for difference (η^2^) was calculated with the formula $\frac{{t}^{2}}{{t}^{2}+(N-1)}$, and was interpreted in accordance with Cohen’s guidelines (small effect size = 0.01–0.05; moderate = 0.06–0.13; large  ≥ 0.14) [67]. Associations between alcohol knowledge (theoretical and practical) and alcohol intervention frequency were explored with bivariate correlation analyses (Pearson *r*) and multiple linear regression analysis. An interaction term (theoretical x practical knowledge) was included in the second step of the alcohol knowledge-intervention frequency analysis in order to explore whether an association between practical knowledge and intervention frequency was moderated by theoretical knowledge, or vice versa. Associations between alcohol knowledge (theoretical and practical) and probability of conducting group and individual interventions were analyzed using non-parametric correlation analyses (Spearman *rho*) and multiple binary logistic regression. The magnitude of variances in the ways of doing (outcomes) explained by the ways of knowing (predictors) and covariates in the regression models (*R*^2^) were interpreted in accordance with Cohen’s guidelines (small = 0.02–0.12; moderate = 0.13–0.25; large   ≥ 0.26) [64].

#### Sensitivity analyses

A series of sensitivity analyses were conducted in order to explore potential clustering effects of OHS personnel being employed in different OHS units. Associations between three unit-level variables (units, geographical location, unit size) and the study predictors (theoretical and practical knowledge) and outcomes (intervention frequency, individual and group interventions) were estimated by means of analyses of variance (ANOVA), chi square tests of independence and independent samples t-tests. Measures utilized in sensitivity analyses are more thoroughly described in Additional file 2.

## Results

### Theoretical and practical alcohol knowledge

A paired-sample t-test revealed that OHS personnel rated their practical alcohol knowledge (*M* = 6.86, *SD* = 2.48) significantly lower than their theoretical alcohol knowledge (*M* = 8.25, *SD* = 1.74), *t* (321) = 12.66, *p* < 0.001. The mean difference was 1.38 units (95% CI 1.17, 1.60) on the scale ranging from 1 to 11 (η^2^ = 0.33), indicating a large difference in accordance with Cohen’s guidelines [67].

### Alcohol knowledge and alcohol intervention frequency

Associations between alcohol knowledge and alcohol intervention frequency are presented in Table 2.

**Table 2:** Associations between alcohol knowledge and alcohol intervention frequency, without (model 1) and with (model 2) interaction between knowledge types

Variable	*r*	Model 1				Model 2
				95% CI for b		
		β	b	Lower	Upper	β
Theoretical knowledge	0.38***	−0.03^ ns^	−0.03^ ns^	−0.13	0.07	0.02^ ns^
Practical knowledge	0.65***	0.60***	0.39***	0.31	0.46	0.71***
Theoretical x practical^A^	0.60***	–	–	–	–	−0.15^ ns^
Sex	−0.01^ ns^	0.03^ ns^	0.11^ ns^	−0.24	0.46	0.03^ ns^
Age	0.23***	−0.00^ ns^	−0.00^ ns^	−0.02	0.02	−0.01^ ns^
Education length	0.18**	0.10*	0.08*	0.00	0.15	0.10*
OHS experience	0.18**	0.06^ ns^	0.01^ ns^	−0.01	0.03	0.06^ ns^
Drinking attitudes	−0.04^ ns^	0.02^ ns^	0.07^ ns^	−0.27	0.41	0.02^ ns^
Ed. nurse^B^	0.25***	0.11^ ns^	0.35^ ns^	−0.09	0.79	0.11^ ns^
Ed. physician^B^	0.25***	0.11^ ns^	0.54^ ns^	−0.04	1.12	0.12^ ns^
Ed. physiotherapist^B^	−0.22***	0.04^ ns^	0.18^ ns^	−0.34	0.70	0.05^ ns^
Ed. other health prof.^B^	−0.23***	−0.05^ ns^	−0.22^ ns^	−0.70	0.27	−0.05^ ns^

Model 1: *R*^2^ = 0.47***

Model 2: *R*^2^ = 0.47***; Δ*R*^2^ = 0.001^ ns^

Results from bivariate correlation analyses and multiple linear regression analyses (N = 322); Dependent variable = alcohol intervention frequency

*r* Pearson correlation coefficient, *β* standardized regression coefficient, *b* unstandardized regression coefficient, *CI* confidence interval, *Ed*. educational background

^A^Interaction term (theoretical x practical knowledge)

^B^Ref. = all other educational backgrounds; ***p < 0.001; **p < 0.01; *p < 0.05

^ns^Non-significant (*p* ≥ 0.05)

As shown in Table 2, both theoretical and practical alcohol knowledge were positively correlated with alcohol intervention frequency (*r*_theoretical_ = 0.38, *p* < 0.001; *r*_practical_ = 0.65, *p* < 0.001), indicating that higher levels of knowledge were associated with higher intervention frequency. The multiple linear regression analysis explained 47% of the variance in intervention frequency (*R*^2^ = 0.47, *p* < 0.001). Practical knowledge (b = 0.39, β = 0.60, *p* < 0.001) made a strong and significant contribution to the model, while theoretical knowledge did not (b = −0.03, β = −0.03, *p*  ≥ 0.05). Inclusion of the interaction term (theoretical x practical knowledge; β = −0.15, *p* ≥ 0.05) suggested that the two ways of knowing did not interact in their association with intervention frequency (Δ*R*^2^ = 0.001, *p* ≥ 0.05).

### Alcohol knowledge and intervention levels

Associations between alcohol knowledge and conducting interventions on individual and group levels are presented in Table 3.

**Table 3:** Associations between alcohol knowledge and intervention levels (individual and group)

	Logistic regression models		Correlations	
	Individual interventions^A^	Group interventions^A^	Individual interventions^A^	Group interventions^A^
Theoretical knwl^B^	OR = 0.92^ ns^ (0.74, 1.13)^E^	OR = 0.80^ ns^ (0.64, 1.00)^E^	*rho* = 0.27***	*rho* = 0.19**
Practical knwl^B^	OR = 1.60*** (1.33, 1.90)^E^	OR = 1.84*** (1.50, 2.26)^E^	*rho* = 0.48***	*rho* = 0.44***
Sex^C^	OR = 1.43^ ns^	OR = 1.17^ ns^	*rho* = 0.07^ ns^	*rho* = 0.02^ ns^
Age^B^	OR = 0.99^ ns^	OR = 0.99^ ns^	*rho* = 0.16**	*rho* = 0.14*
Education length^B^	OR = 1.11^ ns^	OR = 1.01^ ns^	*rho* = 0.10^ ns^	*rho* = 0.03^ ns^
OHS experience^B^	OR = 1.01^ ns^	OR = 1.01^ ns^	*rho* = 0.010^ ns^	*rho* = 0.07^ ns^
Drinking attitudes^B^	OR = 0.61^ ns^	OR = 0.70^ ns^	*rho* = −0.09^ ns^	*rho* = −0.07^ ns^
Ed. nurse^D^	OR = 4.06**	OR = 1.92^ ns^	*rho* = 0.35***	*rho* = 0.22***
Ed. physician^D^	OR = 3.27^ ns^	OR = 1.83^ ns^	*rho* = 0.18**	*rho* = 0.12*
Ed. physiotherapist^D^	OR = 1.25^ ns^	OR = 1.17^ ns^	*rho* = −0.20***	*rho* = −0.16**
Ed. other health prof.^D^	OR = 0.46^ ns^	OR = 0.84^ ns^	*rho* = −0.30***	*rho* = −0.16**
Nagelkerke *R*^2^	0.45	0.36		
Cox-Snell *R*^2^	0.32	0.22		

Results from multiple logistic regression analyses and bivariate (non-parametric) correlation analyses (N = 322)

*OR* odds ratio, *rho* Spearman rho correlation coefficient, *knwl* knowledge, *Ed.* educational background

^A^No intervention activity is the ref

^B^Higher score indicates higher value

^C^Males are the ref

^D^Ref. = all other educational backgrounds

^E^95% confidence interval for OR; *p <0 .05; **p < 0.01; ***p < 0.001

^ns^Non-significant (*p* ≥ 0.05)

As shown in Table 3, bivariate correlation analyses indicated that both theoretical and practical alcohol knowledge were positively associated with conducting individual- and group-level interventions, suggesting that higher levels of knowledge were associated with a tendency to conduct (rather than not conduct) interventions (*rho*_theoretical-individual_ = 0.27, *p* < 0.001; *rho*_theoretical-group_ = 0.19, *p* < 0.01; *rho*_practical-individual_ = 0.48, *p* < 0.001; *rho*_practical-group_ = 0.44, *p* < 0.001). Compared with theoretical knowledge, practical knowledge displayed stronger bivariate relationships with conducting both individual- and group-level interventions.

The individual-level multiple logistic regression model was statistically significant, χ^2^ (11, N = 322) = 123.38, *p* < 0.001, and explained between 32% (Cox and Snell *R*^2^) and 45% (Nagelkerke *R*^2^) of the variance. Adjusted for all other variables in the model, conducting individual-level interventions was more likely when the level of practical alcohol knowledge was higher (for each increasing unit on the practical knowledge scale ranging from 1 to 11, the odds increased by a factor of 1.60; OR = 1.60, *p* < 0.001). Theoretical knowledge was not significantly associated with the odds of conducting individual-level interventions (OR = 0.92, *p* ≥ 0.05).

The group-level multiple logistic regression model was significant, χ^2^ (11, N = 322) = 81.79, *p* < 0.001, with an explained variance of between 22% (Cox and Snell *R*^2^) and 36% (Nagelkerke *R*^2^). Higher levels of practical knowledge were associated with increased odds of conducting group-level interventions (OR = 1.84, *p* < 0.001). A one unit increase in practical knowledge was associated with increased odds of conducting group-level interventions equivalent to a factor of 1.84. Theoretical knowledge was not significantly associated with odds of conducting group-level interventions (OR = 0.80, *p* ≥ 0.05).

**Fig. 2:**
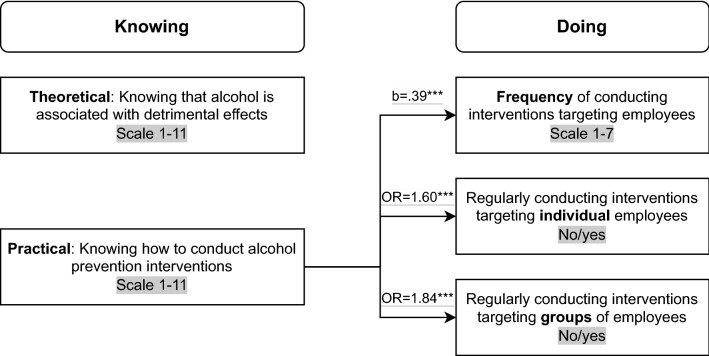
Associations between two ways of knowing (alcohol-related knowledge) and three ways of doing (alcohol prevention interventions)

In summary, associations between the two ways of knowing (alcohol-related knowledge) and the three ways of doing (alcohol prevention interventions) are depicted in Fig. 2.

### Sensitivity analyses

The median number of respondents in each OHS unit was four, rendering it statistically inappropriate to explore differences on the predictors and outcomes between all units. However, analyses of five units with  ≥ 10 respondents (n = 97) indicated that these units did not differ significantly on any of the study variables (theoretical knowledge: *F* (4, 92) = 0.65, *p* = 0.632; practical knowledge: *F* (4, 92) = 0.95, *p* = 0.441; intervention frequency: *F* (4, 92) = 0.24, *p* = 0.913; individual interventions: χ^2^ (4, n = 97) = 1.27, *p* = 0.867; group interventions: χ^2^ (4, n = 97) = 1.77, *p* = 0.778).

Unit location (geographical region) was not significantly associated with the predictors (theoretical knowledge: *F* (4, 321) = 0.49, *p* = 0.742; practical knowledge: *F* (4, 321) = 0.72, *p* = 0.582) or the outcomes (intervention frequency: *F* (4, 321) = 0.23, *p* = 0.924; individual interventions: χ^2^ (4, n = 322) = 2.66, *p* = 0.617; group interventions: χ^2^ (4, n = 322) = 5.88, *p* = 0.208).

OHS personnel in small OHS units (defined by employing 1–7 personnel) reported somewhat more practical knowledge than personnel in large units (employing  ≥ 8 personnel) (*M* = 7.31, *SD* = 2.39 vs *M* = 6.64, *SD* = 2.49, *t* (320) = 2.29, *p* = 0.023). However, unit size was not significantly associated with theoretical knowledge (*M* = 8.47, *SD* = 1.68 vs *M* = 8.13, *SD* = 1.74, *t* (320) = 1.68, *p* = 0.095), intervention frequency (*M* = 3.40, *SD* = 1.55 vs *M* = 3.30, *SD* = 1.63, *t* (320) = 0.53, *p* = 0.600), individual interventions (χ^2^ (1, n = 322) = 0.43, *p* = 0.514), or group interventions (χ^2^ (1, n = 322) = 0.00, *p* = 0.984).

Overall, sensitivity analyses did not indicate the presence of clustering effects of OHS personnel being employed in different OHS units.

## Discussion

The aim of this study was to explore two different ways of knowing about alcohol (theoretical and practical alcohol knowledge) and their associations with three ways of doing alcohol prevention interventions in occupational health settings (intervention frequency, conducting individual and group interventions). Our main findings were: (i) OHS personnel rated their alcohol knowledge quite high, and they rated their theoretical knowledge significantly higher than their practical knowledge, and (ii) only practical alcohol knowledge (not theoretical) was significantly associated with doing alcohol prevention interventions in occupational health settings.

In contrast to earlier studies implicating quite low levels of alcohol-related knowledge among health care personnel [49–54], OHS personnel in our study believed that they were quite knowledgeable about alcohol-related topics. Evidence of limited knowledge among health care personnel has been found regarding topics such as effects of alcohol consumption on health [52], prevalence of alcohol use and risky drinking [49, 53], etiology of alcoholism [54], fetal alcohol spectrum disorders and maternal alcohol use [50–52], drinking limits, guidelines and how to advise patients about responsible drinking [55, 56], how to screen for substance misuse [59], and effectiveness of brief alcohol interventions [53, 54].

OHS personnel rated their practical alcohol knowledge significantly lower than their theoretical knowledge. Practical knowledge (knowing how) is closely related to abilities [45, 46], which may indicate that level of practical knowledge to some extent reflects the degree to which personnel are actually involved in conducting interventions. Earlier research from Norway has indicated that intervention activity in OHS is quite limited [35], which may explain why practical knowledge was rated lower than theoretical knowledge in our study. Theoretical knowledge (knowing that), on the other hand, may stem from a wider range of available sources, such as education, media, literature and so on.

Paradoxically, practical knowledge being the way of knowing rated lowest, was the most important predictor of doing. Practical knowledge was associated with how often OHS personnel conducted alcohol interventions, as well as whether or not they regularly conducted interventions on individual and group levels. Theoretical knowledge was not significantly associated with either. Moreover, there was no evidence of an interaction between the two ways of knowing in their association with doing. Thus, our findings are in line with Holmqvist et al. [57], who studied OHS personnel in Sweden, and found that knowledge about counseling techniques (a type of practical knowledge) was the most important facilitator for increased intervention activity. With expectancy-value theory [47] as a backdrop, self-assessed ability to conduct alcohol interventions (i.e., practical knowledge) may be pivotal in determining positive or negative expectancies which, in turn, may increase or decrease the likelihood of conducting interventions. One could have expected that theoretical knowledge would have played a role in explaining the doing of interventions, e.g., by shaping subjective values toward interventions. However, we did not find such a relationship. It may be that OHS personnels’ values can be understood in terms of factors beyond those measured in this study, or perhaps subjective values are influenced by a wider conceptualization of theoretical knowledge than what has been applied in this study. For instance, an earlier study from Norway found that the majority of OHS personnel perceived that employees’ alcohol consumption constitutes a public health challenge, and that OHS should obtain a more proactive role in alcohol intervention [35]. Moreover, we cannot rule out that the lack of associations between theoretical knowledge and doing alcohol prevention interventions may have been a result of a ceiling effect—the variability in theoretical knowledge was relatively low and, on average, the sample rated themselves particularly high on theoretical knowledge.

According to Fantl [46], theoretical and practical knowledge may be conceived as either independent or interwoven. Our study provides support for the notion that the two ways of knowing are fundamentally independent. Practical knowledge predicted doing interventions independently from theoretical knowledge, and we found no evidence of an interaction between the two ways of knowing in their influence on doing.

### Methodological considerations

Certain limitations should be kept in mind when interpreting results from this study. First, although one may argue that knowledge is more fundamental than overt behavior, reversed or two-way causality cannot be ruled out. For instance, practical knowledge may have determined the extent to which OHS personnel conduct interventions, but the extent to which OHS personnel have conducted interventions may also have had an effect on their practical knowledge. The cross-sectional design of this study allowed us to explore associations between variables, but precluded causal inferences.

Second, theoretical and practical knowledge constitute complex concepts that are difficult to operationalize and measure. In this study, we utilized a quite narrow understanding of these concepts by focusing on theoretical knowledge as participants’ perceptions of the extent of knowing that alcohol is associated with detrimental health and occupational outcomes, and practical knowledge as knowing how to conduct alcohol interventions. Future research should focus on the development of adequate measures, better capable of addressing the complexities of both ways of knowing.

Third, we measured knowledge by means of self-ratings, which may be vulnerable to bias. Although self-ratings of knowledge are widely used in the literature, research is inconclusive on the matter of how valid and reliable such measures are. Some authors [65, 66] advocate that individuals generally have accurate perceptions of their knowledge, while others disagree [67]. Still, reporting bias will be equally problematic (or non-problematic) for both types of knowledge. Although studies have shown that single-item measures can demonstrate satisfactory reliability and validity [68], our measure of practical knowledge was particularly limited due to being measured with a single item as well as being self-rated.

Fourth, the sample size was considered adequate for the intended analyses (N_necessary_ = 180; N_actual_ = 322), and the final response rate was 53.6%. However, a considerable number of OHS units did not respond or did not agree to participate in the study (n = 137 units). Thus, a substantial number of individual OHS personnel in Norway were not reached by our survey, and we did not possess any information about these individuals. As a result of not all OHS units providing us with access to their personnel, the sample was not randomly selected from the population. Moreover, 33 OHS personnel were excluded due to not responding on all relevant study items. However, there were no statistically significant differences in terms of sex, age and OHS experience between those completing and not fully completing the survey [see Additional file 3]. Consequently, some caution is warranted when generalizing the study results. On the other hand, units from all geographical counties in Norway and units providing services for companies across work divisions were represented among the 69 units. Moreover, comparisons of OHS personnels’ educational background in the study sample and in a Norwegian national official evaluation of OHS from 2016 [69] revealed no significant differences, with the exception of physiotherapists being somewhat overrepresented in our study [see Additional file 4]. Although the sex distribution was skewed in our sample (females = 79.2%), it largely reflects the true sex distribution among personnel in health and social services in Norway (females = 82.9%) [70].

### Implications

Our findings suggest that efforts to bridge the know-do gap should focus on maximizing practical rather than theoretical knowledge. For instance, alcohol training programs for OHS personnel, in educational as well as in workplace contexts, should emphasize knowledge about how to deal with alcohol-related issues and how to conduct alcohol interventions in occupational health settings, rather than solely or primarily focusing on knowledge about detrimental effects of alcohol consumption. Even though practical knowledge emerged as the most important predictor of doing, the importance of theoretical knowledge should not be undermined. In addition to actually conducting interventions, it is important that health care personnel know why interventions are done, the evidence they are built upon, and how they work. Hence, training programs should aim for a serviceable integration of the two ways of knowing, but with an emphasis on practical knowledge. Sensitivity analyses did not indicate the presence of clustering effects, implying that potential contextual differences across OHS units did not affect associations between knowing and doing on an individual level. This finding strengthens the rationale for targeting individual OHS personnel’s knowledge, while still encouraging unit policies and practices supportive of more focus on training programs emphasizing practical knowledge.

Our data were collected in 2018, prior to the outbreak of the COVID-19 pandemic. Some studies suggest that the COVID-19 pandemic made it necessary for OHS to somewhat modify their work patterns, e.g., in terms of employing extra staff, offering out-of-hours and weekend services, and prioritizing certain tasks such as vaccination and sick leave surveillance [71, 72]. In some cases, this shift may have resulted in OHS being less present at worksites, e.g., by reducing their number of workplace visits [73]. However, a survey among occupational physicians in the UK [72] found that the majority of OHS units (93%) continued to offer routine services during the pandemic. The prevalence of teleworking (working outside conventional office settings) increased tremendously during the pandemic and will likely, to some extent, remain a routine practice for employees throughout the progression and cessation of the pandemic [74]. Increased teleworking among employees may pose a dual challenge for OHS' prevention activities: Employees may become less visible and accessible to OHS personnel, and some studies have indicated that teleworking is associated with increased alcohol consumption [75]. Hence, post-pandemic work dynamics may necessitate innovative measures in OHS' alcohol prevention activity, for instance in terms of increased application of digital platforms.

A venue for future research is to develop, implement and test alcohol training programs tailored for OHS personnel. Such research should include effect studies as well as implementation studies. Although our regression models largely explained variances in the behavioral outcomes (between 22 and 47%), a considerable amount of the variances in doing was not explained by our models focusing on knowledge. Hence, further research on potential antecedents and correlates of conducting interventions is warranted. Furthermore, future research should explore associations between different types of knowledge and intervention activity with regard to other health and risk behaviors (e.g., healthy diet, physical exercise, smoking and drug use).

## Conclusions

This study demonstrated that different ways of knowing about alcohol among OHS personnel were dissimilarly associated with conducting alcohol prevention interventions targeting employees in occupational health settings, and that efforts aimed at bridging the know-do gap would benefit from focusing on practical rather than theoretical knowledge. Stated differently, for doing, knowing how is more important than knowing that. Training programs for OHS personnel should emphasize knowledge about how to deal with alcohol-related issues and how to conduct prevention interventions.

## Supplementary Information

**Additional file 1. **Component structure and internal consistency for the alcohol knowledge items.

**Additional file 2. **Descriptions of measures utilized in sensitivity analyses.

**Additional file 3. **Comparisons between completers and non-completers.

**Additional file 4. **Study selection analysis.

## Data Availability

The datasets used and analyzed during the current study are available from the corresponding author on reasonable request.

## Abbreviations

ANOVA: Analysis of variance
knwl: Knowledge
M: Mean
NSD: Norwegian Centre for Research Data
OHS: Occupational health services
OR: Odds ratio
SD: Standard deviation
WIRUS: Workplace interventions preventing risky alcohol use and sick leave
