# Supplementary material for: Working with alcohol prevention in occupational health services: “knowing how” is more important than “knowing that” – the WIRUS OHS study
Source: Addict Sci Clin Pract. 2022 Oct 1;17:54. doi: 10.1186/s13722-022-00335-0 (PMC9526525; doi:10.1186/s13722-022-00335-0)
Supplement: Supplementary file 1 — Additional file 1. Component structure and internal consistency for the alcohol knowledge items. [file 13722_2022_335_MOESM1_ESM.pdf]

Additional File 1. Component structure and internal consistency for the alcohol knowledge items

	Pattern matrix		Structure matrix		
Item	F_1	F_2	F_1	F_2	Communality
(T) Alcohol – sick leave	**0.93**	0.03	**0.94**	0.60	0.89
(T) Alcohol – presenteeism	**0.91**	-0.02	**0.90**	0.54	0.81
(T) Alcohol – health	**0.89**	-0.01	**0.89**	0.54	0.79
(P) Alcohol prevention	0.00	**1.00**	0.62	**1.00**	1.00

	F_1	F_2	Both
Eigenvalue λ (% explained variance)	2.96 (74.10)	0.52 (12.98)	(87.09)
Cronbach's α	0.89	-	0.87
Mean inter-item correlation	0.74	-	0.65

Component structure generated with confirmatory principal component analysis applying oblique rotation; Kaiser-Meyer-Olkin measure of sampling adequacy (KMO) = 0.80; Bartlett's test of sphericity p <.001; T = a priori theoretical knowledge item; P = a priori practical knowledge item
