# Supplementary material for: Working with alcohol prevention in occupational health services: “knowing how” is more important than “knowing that” – the WIRUS OHS study
Source: Addict Sci Clin Pract. 2022 Oct 1;17:54. doi: 10.1186/s13722-022-00335-0 (PMC9526525; doi:10.1186/s13722-022-00335-0)
Supplement: Supplementary file 2 — Additional file 2. Descriptions of measures utilized in sensitivity analyses. [file 13722_2022_335_MOESM2_ESM.pdf]

Additional File 2. Descriptions of measures utilized in sensitivity analyses

Due to potential clustering effects of OHS personnel being employed in different OHS units (i.e., the potential of findings being attributable to differences between units rather than differences between personnel), three unit-level variables were utilized in sensitivity analyses. First, personnel were grouped within their units (a total of 69 units, of which 5 consisted of ≥10 responding personnel). Second, personnel were grouped in accordance with their OHS units' geographical location (Eastern Norway, n = 148; Western Norway, n = 76; Mid-Norway, n = 36; Southern Norway, n = 36; Northern Norway, n = 26). Third, units were dichotomized into small vs large units (split by the median value of seven eligible personnel in each unit; respondents in small units: n = 107, large: n = 215).
