# Supplementary material for: Working with alcohol prevention in occupational health services: “knowing how” is more important than “knowing that” – the WIRUS OHS study
Source: Addict Sci Clin Pract. 2022 Oct 1;17:54. doi: 10.1186/s13722-022-00335-0 (PMC9526525; doi:10.1186/s13722-022-00335-0)
Supplement: Supplementary file 3 — Additional file 3. Comparisons between completers and non-completers. [file 13722_2022_335_MOESM3_ESM.pdf]

Additional File 3. Comparisons between completers and non-completers

Variable	Completers[A]	Non-completers[B]	*p*
Sex, % females	79.2	86.7	.329[C]
Age, *M*	48.9	46.0	.122[D]
OHS experience, *M*	12.0	9.9	.208[D]

[A]OHS personnel who responded on all study items, i.e., the study sample (n = 322); [B]OHS personnel who consented to participate but did not respond on all study items (n = 33); [C]Difference tested with chi square test of independence; [D]Difference tested with independent samples t-test
