# Supplementary material for: Working with alcohol prevention in occupational health services: “knowing how” is more important than “knowing that” – the WIRUS OHS study
Source: Addict Sci Clin Pract. 2022 Oct 1;17:54. doi: 10.1186/s13722-022-00335-0 (PMC9526525; doi:10.1186/s13722-022-00335-0)
Supplement: Supplementary file 4 — Additional file 4. Study selection analysis. [file 13722_2022_335_MOESM4_ESM.pdf]

Additional file 4: Study selection analysis

**OHS professionals' background**	Study sample (N = 322), % (n)	Mandal et al. (2016) (N = 766), % (n)	Difference	
			Percentage points	*p* value
Nurse	37.9 (122)	42.0 (322)	4.1	.209[ns]
Physiotherapist	17.7 (57)	9.4 (72)	8.3	<.001***
Physician	13.0 (42)	11.1 (85)	1.9	.373[ns]
Occupational therapist	3.1 (10)	3.8 (29)	0.7	.571[ns]
Occupational hygienist	8.4 (27)	7.7 (59)	0.7	.696[ns]
Psychologist	1.9 (6)	2.1 (16)	0.2	.831[ns]
Nutritionist	0.3 (1)	1.0 (8)	0.7	.235[ns]†

[a]Mandal R, Dyrstad K, Melby L, Midtgård T. Evaluering av bedriftshelsetjenesten i Norge [Evaluation of the occupational health services in Norway]. Oslo, Norway: Sintef; 2016; Differences tested with chi-square tests of independence; †Difference tested with Fisher's exact test; ****p* <.001; [ns]Non-significant ($p \geq .05$)
